# Autism as emergent and transactional

**DOI:** 10.3389/fpsyt.2022.988755

**Published:** 2022-10-07

**Authors:** Jonathan Green

**Affiliations:** Division of Psychology and Mental Health, Faculty of Biology Medicine and Health, Manchester Academic Health Sciences Centre, Royal Manchester Children’s Hospital, University of Manchester, Manchester, United Kingdom

**Keywords:** autism, transaction, emergence, neurodiversity, intervention, autism spectrum conditions, clinical trials, neurodivergence

## Abstract

The current epistemology of autism as a phenotype derives from the consistency of historical accounts and decades of work within the tradition of descriptive epidemiology, culminating in current categorical descriptions within DSM and ICD nosologies and the concept of “prototypical autism.” The demonstrated high heritability of this phenotype has led to an essentialist theory of autism as a biological entity and the concerted search within the developmental brain and genetic science for discrete biological markers. This search has not revealed simple markers explaining autistic outcomes and has led to moves towards a more dimensional account. This article proposes an alternative transactional approach. It proposes to understand autistic states as an emergent property within a complex developmental system; as the neurodivergent brain, and mind and body, encounter their social and physical environment within early development. Key evidence in support of this approach comes from random allocation intervention trials based on such transactional development theory, both in the infancy pre-diagnostic prodrome and the early post-diagnostic period. In replicated evidence, these intervention trials show that a targeted alteration in the quality of social transactional environment available for the child leads to significant, predictable, and sustained alterations in the outcome dimensional autistic phenotype over time; and further, in one prodromal trial, to a significant reduction in later categorical classification status. The inference from this evidence is that the prototypical autistic phenotype is to a degree malleable with a changed experienced social environment and that it is emergent from its constituent traits. Such a transactional approach enlarges our notion of the phenotype and brings the study of autism within mainstream individual difference developmental science. It challenges essentialist views, for instance as to intrinsic autistic “social avoidance” or theory of mind empathy deficits, integrates dimensional and categorical perspectives, and is consistent with the lived experience of autistic people and their advocacy for improved understanding within a social model.

## Introduction

### Dimensional and categorical autism

One of the original tenets behind the National Institutes of Health Research Domain Criteria project (RDoC),^1^ an initiative in relation to neurodevelopmental conditions, well-advocated by Insel (1, 2), was the aspiration to replace current nosological behavioural phenotypes with the antecedent neurodevelopmental trajectories underpinning them (3). However, this impetus finds additional weight from different sources too; for instance, from many in the developmental psychology and research community who instinctively lean towards “dimensional” approaches to development and psychopathology, and in recent advocacy from many in the autistic community. The idea of categorical autism has sometimes come to be equated negatively with what can be felt as a reductive “medical model,” with implicit associations to the experience of unequal power relationships in clinical practice, academia, and social life. These are delicate waters. On the one hand, there are key strengths in the dimensional approach, which is in many ways fundamental to what I will be arguing in this article in terms of a transactional account of neurodiversity development within the social sphere. On the other hand, I wish to argue that opposing dimensional and categorical accounts in this way sets up a false binary. The dimensional and the categorical lenses have always been present in our developmental thinking—and both are crucial. It is intrinsic to our perceptual and cognitive functioning that we look at both process *and* entity as complimentary; it is the wood *and* the trees. The term “medical model” can sometimes underplay the sophisticated underpinnings of developmental psychopathology and psychiatry, not to mention good clinical work. This article is written from that clinical science tradition—indeed, aspiring to update that tradition into the current context.

There are also paradoxes in a purely “dimensional” approach. One of the immediate paradoxes is sure that the very term “autism” is categorical; a term that has been used historically to name something, and has also more recently become a term naming a valued social identity. The history and evolution of this naming is in itself a valuable subject for reflection (4); from the earliest highly theory-driven accounts of Bleuler and others, the more considered clinical descriptions of Sukhareva (5), Binswanger, and Kanner; into the tradition of empirical description and nosology elaborated in the last 60 years, which morphed into the developmental science and neurodevelopmental account of the current paradigm. The rise of the social advocacy and the pressing forward of social identity in relation to autism introduces a new note into this progression; a lived-subject assertion of experience which results in a rather different idea of autism as an “identity”—giving, in Levi-Strauss’s formulation, “every individual … his own (identity) as …a signifier of his signified being” (6). An easy momentum from now could be towards the term “autism” fragmenting, and becoming applied quite differently to a prototypical phenotypic description for researchers, a diagnostic construct for clinicians and health service administrators (for instance as a ticket to a service support), and a social identity signifier within the community. Lack of mutual understanding across these domains would inevitably grow. Some may feel such fragmentation is inevitable or indeed desirable, and there are, indeed, deep differences between some of these perspectives. However, the implicit aim of clinical science towards evidence-based practice over decades has been to bridge these domains; working with research and in dialogue towards a stable descriptive language that could integrate science, evidence-based clinical practice, and social understanding. There are opportunities within current challenges and debates to enrich and develop this common language with new insights, and to reduce misunderstanding; this article is part of trying to do just that.

Further, in scientific terms, replacing categorical autism with a neurodiversity dimension just replaces one complex paradox with another. Decades of neurodevelopmental science have not yet succeeded in defining a commonly accepted neurodevelopmental trajectory unique to autism (7, 8), and in this context, the RDoC project is far from realising success (9). An alternative strategy has been to replace the notion of an autism phenotype altogether with a series of RDoC-inspired “transdiagnostic” trait phenomena (such as impulsivity, executive function) and to make these targets for attention and intervention as “needs” rather than autism itself (10, 11). But this deconstruction of the autistic phenotype has been criticised by Mottron et al. (12) as potentially leading to a series of false equivalents or homologues; those specific phenomena may appear superficially similar but actually be very different in different contexts. Without care and accurate demonstration of real equivalence, such an approach would threaten to collapse nuance and discrimination in developmental science. Advanced machine learning and deep learning paradigms *may* provide a route into an alternative empirical way of moving from observed traits to an autism entity (10); however, there are no reproducible outcomes from this as yet. The strategy will also depend, just as previous research has done, on the quality of the basic measurements that are undertaken. In many ways, the proposed path to automation will need to parallel the methodological efforts from previous decades of clinical observation, clinical practice, and developmental science research; it will also itself have to wrestle with these same paradoxes of dimension vs. category in development.

### Combining categorical and dimensional accounts: Autism as “emergent”

In sum, the pitting of dimensional against categorical remains, as it always has been, a false binary. This article proposes to cleave, for historical, linguistic, conceptual, and pragmatic reasons at this point, to the notion of a prototypic autism entity articulated by Mottron (12), but to avoid false binaries by seeing the autism entity of this kind as an *emergent property* within complex-system neurodevelopment—where “emergent” is stringently defined as referring to “arising phenomena that are novel and that differ in type and quality from the interacting components” (13, 14). This is a more dynamic model which defines autistic “states” arising out of dimensional variation, rather than pre-formed entities. It combines constitutional difference, transactional experience, and phenotypic entity into a mutually informing whole (15–17). There is an acknowledged challenge (13) in translating such appealing metaphor and theory into the operational description and the investigative strategies necessary to do science, for instance, to formulate testable and refutable hypotheses (18); empirical success to date with dynamic system modelling has largely been restricted to motor development in children, with analogies made to wider aspects of development (17). The strategy I will take towards this is to focus on the key moments of emergence and subsidence of the phenotype as points of entry for understanding; particularly focusing on the insights that can be gained from investigative experimental clinical trials; using the classic approach that a good way towards the understanding of a complex system is by trying to change it.

## Approaching autism emergence and epistemology through empirical trials

There are three ways of addressing empirically a notional property of emergence: (i) constructing an observational account of phenotypic emergence within early development (12), (ii) observation of any possible phenotype subsidence later in development (19), and (iii) the effects of an experimental intervention into developmental processes through randomised allocation clinical trials. The first two can only essentially be approached through clinical description or longitudinal observational paradigms. These can be highly informative but are subject to a range of confounds that can limit the strength of inferences. The third, however, because it is the result of a controlled test of the results of a discrete and well-characterised developmental change, provides the most robust way into causal inference for the phenomenon of emergence, and that is what I focus on here.

### Outcome measurement

In a series of investigative randomised controlled clinical trials, myself and colleagues have been able to address questions of emergence in this “experimental” sense. This was possible because we included in the initial design of these trials from 2000, as our pre-specified primary outcome, a specific measure of the autism phenotype [Autism Diagnostic Observation Schedule (ADOS; 20) or Autism Observation Scale for Infants (AOSI; 21)]. This was done at that time from an assertion that any intervention that wished to claim effect on “autism” should have as its outcome some measure of the actuality of “autism” itself, rather than solely some proxy or parallel measure of adaption or other functioning. This choice in itself is a deep issue for epistemology; it implies that a scientific understanding of the phenotype is only possible through measurement and that the form of measure chosen needs both accurately to reflect the richness of the phenomenon or phenotype in question and to be fit for the purpose for which it is being used. Measures of a complex reality like autism have a hard challenge to translate the complexity into quantifiable data for analysis. For our use, the ADOS, in particular, had the strong advantage of being the best-validated proxy for the full range and richness of the formal phenotype, with extensive psychometric and longitudinal cohort work behind it (20); and also being objectively and reliably codable from videotape, thus allowing blinded ascertainment and reduced bias for a trial. Further, the development of developmentally specific ADOS modules (22) facilitated the comparability of measurement over development and time, crucial in facilitating the longitudinal study of the clinical phenotype through the differing presentations as development proceeds and allowing our follow-up studies of trial outcomes.

Much has changed in the dialogue around autism in recent years and, within that, ADOS has been criticised (along with related nosological phenotypic definitions) for its normative and “deficit-focused” assumptions (23). Alternative measurement innovations have been proposed that aim to make less normative assumptions (24). These new approaches are in their early stages and may, indeed, prove transformative as they develop, but in the meantime, we need to address the concerns around the ADOS and the value of the corpus of results from it. I acknowledge the concerns but point to the roots of ADOS as a distillation of the clinical encounter; in good hands, it is sensitive to the autistic child and can bring out their ability across a range of social contexts, allowing the manifestation of both strengths and difficulties within autistic difference. When I administer it I feel I am able to engage deeply with a key part of the child’s personhood and development. The language used in the coding may now seem over-medicalised and deficit-focused and this could be usefully updated without affecting the essence of what it does. No measurement is perfect but the ADOS I would say remains the best current consensus means of measuring the observed behavioural phenotype and I hope to be able to demonstrate below the richness and power of what it can tell us. A particular unknown when we started was whether we would be able to show any intervention-related change in an instrument that was essentially designed as a stable phenotypic measure. In the event, throughout the programme to be described, both ADOS and AOSI have proved informative measures that are sensitive to intervention-related change. In a later discussion of these results, I will discuss more fully the nuances and caveats as to what we can learn from this measurement and try to address potential misunderstandings. I also note a key part of what is missing in current measurement—phenomenology—and point the way forward to a key new area of measurement practice.

### Post-diagnostic intervention

The intervention used in this programme, called the Paediatric Autism Communication Therapy (PACT), was specifically designed to address the early developmental precursors of social communication, social engagement, and language relevant to the autistic phenotype and its emergence. The therapy was initially designed for the pre-school period. It works with parents in a naturalistic context aided by video-feedback techniques to help their awareness and understanding of the particularities of the communication style and intent of their neurodivergent child; in consequence improving the accuracy, sensitivity, and contingency of their dyadic responses. The model is that the young autistic child will then respond in turn with increased social response and communication initiation. What emerges is a powerful “coupling” of social interaction (25) of a kind that is central to naturalistic social learning in development (26). In this approach, there is no direct therapeutic work with the child, the focus is on the surrounding interpersonal and communication context. Any alteration in child behaviour, social orientation, and motivation comes naturalistically as a by-product of the altered dyadic response from the parent in real-time. Such an approach can be distinguished from traditional behavioural learning models of therapy such as EIBI or ESDM, either delivered by the therapist or the parent, which target specific behaviours to change in a specified direction through operant conditioning with rewards and contingency reinforcers. PACT therapy is manualised and developmentally staged to build on this early dyadic synchrony towards further social and communication engagement. In the trial testing, the extent to which the parent is able successfully to understand and respond to the child in this way is measured through an assessor-blinded coding of the proportion of parental “synchronous responses” within a video-sample of parent–child free play taken separately to the therapy context. Similarly, the extent to which the child responds is coded through the proportion of their behaviours that are “social communication initiations” to the parent. These alterations in the dyadic interaction between parent and child are the proximal target of the therapy, with the developmentally based hypothesis that such change will translate through a developmental cascade into more “distal” generalised improvement in child functioning in different contexts and through time as they grow. In our trials, this more generalised improvement later in time is measured with another adult in the context of the ADOS assessment, as well as more functional parent and teacher-rated outcomes. Such style of measurement thus allows a precise mechanism testing of the logic model of the therapy, since the developmental hypothesis predicts a cascade of effects from parent synchrony to child initiation to generalised enhancement of the child’s social engagement and communication beyond the dyadic context. The “distal” effect on the phenotypic expression, measured by the ADOS, is thus the pre-specified primary outcome test in the trial.

**FIGURE 1:**
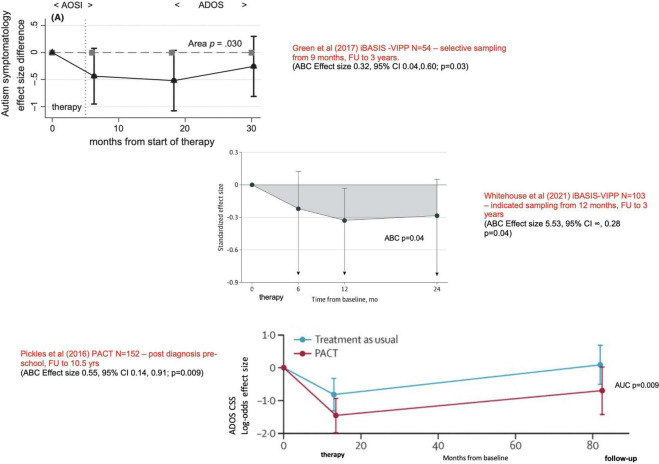
Long term developmental outcomes from three parent-mediated intervention trials. Showing replicated effects at different stages in early development to improve a combination of social communication skills, behavioural rigidity, and sensory sensitivities; improvements sustained after the end of therapy. Reproduced from Green et al. (43), Whitehouse et al. (44), Pickles et al. (29)–see main text. ABC/AUC, Area between curves estimation over time. These estimates provide a principled basis for an overall mean effect for unequally spaced measures that summarise treatment effect over the whole trial from baseline to follow-up.

The first randomised controlled trial (RCT) of this intervention compared to usual care (27) found a substantial treatment effect on the outcome child ADOS total score [*F*_(1,25)_ = 7.30; *p* = 0.01], a result particularly carried by therapy effect to increase function in the “social communication” domain as it was then called. In the subsequent larger PACT RCT (28), we found at the treatment endpoint point a trend for positive intervention effect on both social communication and “restricted repetitive behaviour” including sensory (RRB) domains of ADOS considered separately, and when these were considered together as the full autistic phenotype (29), they showed a significant endpoint treatment effect to reduce the (dimensional) ADOS “combined severity score” (CSS) (OR −6.4; −1.22, −0.06, *p* = 0.02). This endpoint treatment effect was then shown to be sustained; in follow-up intention to treat analysis 6 years after treatment end, 80% of the original cohort of children were assessed at a mean age of 10.5 years, with the assessors remaining blinded to the originally allocated groups. The analysis showed that the between-groups treatment effect on ADOS scores continued all through this time (OR −8.2; −1.53, −0.12, *p* = 0.02), giving a highly significant cumulative effect of the therapy [marginal log-odds effect size of 0.55 (95% CI 0.14–0.91; *p* = 0.009)] (29; see Figure 1). This kind of cumulative analysis is important in giving insight into the ongoing impact of an intervention on development. While these ADOS outcomes were the nominated primary outcome of the trial, effects supporting this change were also seen in parent-reported outcomes in relation to communication, adaptation, and family functioning; teacher ratings of adaptive function in school. One area not showing change was objectively measured “structural” language (vocabulary and grammar) despite parent accounts of vocabulary and communication increasing.

### Process of intervention effect

**FIGURE 2:**
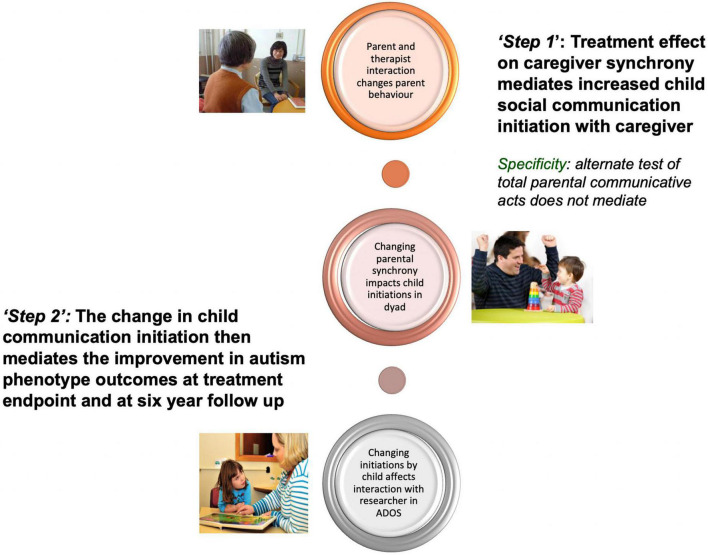
Mediation analysis of the PACT trial (31, 32).

Mechanistic analysis of both these trials identified the mediating (or “active”) processes at different stages of the therapy towards achieving these outcomes. In the first trial, the increased parental synchrony from treatment mediated the ADOS change at the end of therapy (30). In the second larger trial, a two-stage process was identified (31): in the *first step* within the immediate “proximal” parent–child dyad, increased parental synchrony strongly mediated the improvement in child communication initiation with a parent. Then in a *second step*, that improvement in child dyadic communication in turn strongly mediates the later improvement in ADOS generalised outcome. In a further result, it was these same improvements in child dyadic communication initiation during the intervention period that also strongly mediated the sustained reduction in ADOS severity score from endpoint through to 6-year follow-up in middle childhood (32; Figure 2). We thus here identify two stages also in the timing of effects: The first immediate short-term effect on the dyadic interaction of increased parental understanding, responsivity, and “synchronous” communication is to increase the child’s spontaneous social initiation and engagement. This evidences the intended emergent “coupling” of social interaction (25), which is also marked by an increase in manifest shared enjoyment and parent reports of “light-bulb” moments of connectedness (often for the first time) with their child (33). Such a finding is consistent with much of what we know about how dyadic interaction works in neurotypical social development: but what is new here [and consistent with some other intervention research (34)] is to find that neurodivergent children also respond in a similar way, with increased social engagement. This crucial discovery gives empirical evidence counter to an “essentialist” notion of innate unchangeable social avoidance or disinterest in autistic development, suggesting that it is more contextual than that; consistent with a position increasingly advocated in the theoretical literature (35). Then secondly over a longer timescale, we see a “within-child” process that allows the generalisation of the short-term change into longer-term impact on child social communication, behavioural, and adaptive outcomes in development. These longer-term improvements are not so much in formal “structural language” (extent of vocabulary, etc.), which does not change in objective tests, but rather in the pragmatics of social discourse, which does show objective improvement—and this later can be seen as of the key importance, acting as an interactional accomplishment for the child and increasing their connectedness (36). Parent reports of wider development and family effects also suggest broader improvements (37). These mediation results support the logic model of the PACT parent-mediated intervention, as to how it is intended to work. They have further developmental implications for a wider transactional model of autism development, as I will develop further below.

A more recently published trial tested an adaptation of this original clinic-based PACT therapy into a multicomponent intervention simultaneously at home with a parent and in education/school with learning support assistants (PACT-G, 38). This shows both similar and different effects: similar in the significant “proximal” effects of an intervention to improve parental synchrony and child communication in dyadic interaction across all contexts (albeit at a reduced effect size to the original clinic-based PACT trial), different in the lack of transmission and generalisation of these dyadic effects into independent ADOS change. Mechanistic analysis of this PACT-G trial (38) shows a replication of the first stage PACT mediation from parental synchrony to child dyadic communication, but there was a lack of the second stage generalisation process. We put this lack down to the reduced dosage in each context in the PACT-G model, the complexities of implementation in education and also at home, evidence by reduced model fidelity, and possibly the effect of a substantial proportion of online therapy in this iteration. The important learning from this trial is around dosage thresholds and implementation context methods.

### Pre-diagnosis intervention in the autism prodrome

Myself and colleagues then developed a related style of parent-mediated video-aided therapy specifically designed to address the very early infant precursors of prodromal autism. The theory and hypotheses behind this work were similar to that in the post-diagnostic PACT, but the method was adapted to what we knew of early-emerging developmental differences in some infants with a high likelihood of developing later autism, and the empirically observed interaction changes in such groups the first year (39). The therapy manual was adapted from work with neurotypical infants into the briefer 5-month iBASIS-VIPP manualised home-based intervention (40). It is important to note that, as with PACT, there is no intention in this therapy to “change” unwanted child behaviours (concerns that have been expressed in relation to some early intervention strategies). Rather the aim is to increase parental awareness of and sensitivity to neurodivergence in their baby, increasing by this the infant’s experience of being attended to, understood, and responded to by others; and through that to support and nurture the neurodivergent infant’s development and outcomes (41). This is an important point of difference that speaks to the need for promoting autonomous outcomes in early intervention outcome work (42). Results are available from two clinical trials of this parent-mediated intervention on two different populations of infants with an increased likelihood of autistic development. One ascertained through familial incidence (infant siblings of an autistic child) and intervention initiated from mean age 10 months (43), the other with babies identified in community health services at mean age 13 months as having early developmental features suggestive of the raised likelihood of later autism (44). In both these trials, the distal autism phenotypic outcomes were measured as developmentally appropriate using AOSI and ADOS instruments. Both trials showed the sustained impact of intervention on AOSI and then ADOS scores over the 2 years following intervention until diagnostic evaluation at 3 years (see Figure 1). The latter trial (44) additionally had a large enough sample to enable results on a diagnostic evaluation at 3 years, conducted by blinded independent experienced clinicians using clinical best-estimate algorithms from all available information against DSM categorical criteria. This showed a treatment difference across the three categorical autism domains in favour of iBASIS-VIPP therapy (Table 1); amounting to a 60% reduction in emergent autism overall diagnosis at 3 years after intervention (20.5% emergence in TAU against 6.7% in the iBASIS-VIPP group); an odds ratio of 0.18 (0–0.68; *p* = 0.02) or a “number needed to treat” of 7.2 interventions to reduce one autism classification (44). It is important to note here and will be discussed further below, that the children in the therapy group not developing above an autism threshold still did show evidence of other developmental differences of various kinds.

**TABLE 1:** Clinical best estimate ascertainment against categorical DSM5 criteria at 3 years of age, comparing groups receiving iBASIS-VIPP intervention at 1 year and usual care; showing treatment effects on social reciprocity, restricted repetitive behaviours and sensory symptoms (see text).

	No. (%)		Fisher exact test		Binary logistic regression analysis^a^	
Variable	iBASIS-VIPP group (*n* = 45)	Usual care group (*n* = 44)	Odds ratio (95% CI)	*P*-value	Odds ratio (95% CI)	*P*-value
**DSM-5 criterion**						
A1: deficits in social-emotional reciprocity	9 (20.0)	16 (36.4)	0.44 (0–1.08)	0.07	0.35 (0–0.82)	0.02
A2: deficits in non-verbal communicative behaviours used for social interaction	13 (28.9)	17 (38.6)	0.65 (0–1.49)	0.23	0.47 (0–1.08)	0.07
A3: deficits in developing, maintaining, and understanding relationships	13 (28.9)	16 (36.4)	0.71 (0–1.65)	0.3	0.60 (0–1.31)	0.14
B1: stereotyped or repetitive motor movements, use of objects, or speech	7 (15.6)	14 (31.8)	0.40 (0–1.04)	0.06	0.29 (0–0.73)	0.02
B2: insistence on sameness, inflexible adherence to routines, or ritualized behaviour	2 (4.4)	2 (4.5)	0.98 (0–9.40)	0.49	1.03 (0–6.21)	0.51
B3: highly restricted fixated interests that are abnormal in intensity or focus	3 (6.7)	2 (4.5)	1.49 (0–12.57)	0.67	1.16 (0–6.50)	0.56
B4: hyperreactivity or hyporeactivity sensory input or unusual sensory interests	2 (4.4)	8 (18.2)	0.21 (0–0.94)	0.04	0.13 (0–0.53)	0.02
**Diagnosis**						
ASD	3 (6.7)	9 (20.5)	NA	0.07	0.18 (0–0.68)^b^	0.02
Atypical development	37 (82.2)	27 (61.4)	NA	NA	NA	NA
Typical development	5 (11.1)	8 (18.2)	NA	NA	NA	NA

Reproduced from Whitehouse et al. (44).

ASD, autism spectrum disorder; DSM-5, Diagnostic and Statistical Manual of Mental Disorders (Fifth edition); iBASIS-VIPP, iBASIS-Video Interaction to Promote Positive Parenting; NA, not applicable.

^a^The binary logistic regression analysis incorporated the following covariates: infant age at the 24-month postbaseline assessment, baseline score on the Autism Observation Scale for infants, and infant sex.

^b^The binary logistic regression analysis comparing ASD vs. no ASD incorporated the following covariates: infant age at the 24-month postbaseline assessment, baseline score on the Autism Observation Scale for infants, and infant sex.

## Autism as emergent

**FIGURE 3:**
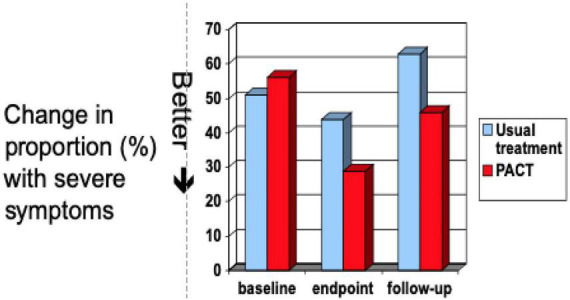
Distribution on dimensional ADOS CSS after post-diagnostic PACT intervention through to 6 year follow up, compared to treatment as usual. Data from Pickles et al (29).

**FIGURE 4:**
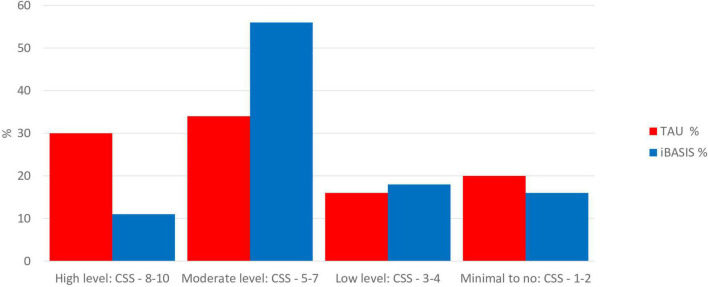
Distribution on dimensional ADOS CSS at 3 years, comparing pre-emptive iBASIS intervention from 1 year with treatment as usual. Data from Whitehouse et al (44).

In sum, there is through this programme, testing a model of intervention targeting developmental precursors of social functioning and autistic development, a consistent replicated pattern of treatment effect across trials on the nosological phenotype as reflected in the ADOS score, sustained in development for several years subsequent to treatment end (Figures 1, 3, 4). Effects are seen at different developmental ages but with the same basic characteristics in response to essentially the same kind of intervention. These effects are seen in relation to the ADOS considered dimensionally, notably across *all* components of the phenotype, both social communication and restricted, repetitive and sensory behaviours. Additionally, in the latest pre-diagnosis trial (44), for the first time, effects are also seen in terms of reducing the incidence of the categorical diagnostic phenotype, as assessed by independent clinicians (Table 1).

The results of these trials taken together represent, therefore, for the first time, a replicated experimental change in autism phenotype emergence/submergence in a way that links together dimensional and categorical approaches. The fact of this replicated effect on dimensional ADOS at different developmental ages makes unsurprising the fact that the intervention also, in the pre-diagnosis trial powered to show this, systematically alters the relation of children to clinical thresholds, such that they no longer meet “autism” criteria (i.e., a prototypical autism description) on best-estimate clinical diagnosis (although they remain neurodivergent). The *threshold categorical* effect (Table 1) is thus directly related to critical changes in *dimensional components* seen in both pre-diagnostic and post-diagnostic cohorts (Figures 1, 3, 4). This is an illustration of the traditional relationship between dimensional change and threshold-related categorical shifts within a complex system. The intervention experiment causes critical planned alterations in the dimensional components, which are linked in turn to whether or not the categorical phenotype emerges. The logical inference from this must be that the categorical phenotype itself is an “emergent” phenomenon; altered threshold effects on ADOS reflect the emergence of a prototypical phenotype from a combination of constituent traits acting within a complex system; a strict definition of emergence (13, 14). Emergent autism in this sense has a particular “quality” that is not manifesting in the constituent traits in children with neurodivergence below the autism threshold. This is in line with the traditional view that the autistic phenotype historically described and evolved does have internal coherence and a predictive and face validity. The described phenotype is not perfect and contains inherent complexity and contradictions, especially as its boundaries have been flexible to changing perceptions; the complexities underlying Mottron’s prototypical suggestion (12). However, it has the virtue of utility and predictability. Efforts to identify an autism-equivalent inductively from constituent traits using for instance machine learning techniques have not so far shown replicable success. The fact remains that, in approaching complex systems, the level of analysis is always crucial—and a level of analysis that includes the historically determined prototypical autistic form has proven its longevity and utility.

What are we to learn then from this about the autism phenotype as measured in this way? I have argued above, with acknowledged caveats, for the veracity of ADOS measurement in reflecting the characteristic breadth and richness of the presentation of autism as behaviour. Our clinical trials data suggest that a consistent, reproducible long-term change in this presentation is possible with targeted early intervention that focuses on the quality of interpersonal and communication environment around the child. The change involves an increase in the child’s social orientation and ability within interaction and communication; also in a reduction in the amount of sensory-related and repetitive behaviours, a “cross-domain” effect across all aspects of the phenotype that is very salient from a theoretical perspective. Equally, however, I would not want to overemphasise the extent of this phenotypic malleability: the amount of difference that we show in these trials is statistically significant but not massive or magical; children in middle childhood after pre-school intervention generally remained autistic and those after infancy intervention who did not develop the emergent phenotype still showed neurodivergent development of other kinds. ADOS results can be confounded sometimes by cognitive ability (45) or clinical heterogeneity (46); although there is no evidence in the trials described above that either of these factors modifies the intervention results reported here. However, these intervention studies do show that the autistic phenotype understood like this is neither fully predetermined nor inviolable; it has empirical malleability to intervention. Nor is it the case that this malleability is confined to “higher functioning” autistic states; the trials described above apply to a range of core (27, 29) to “spectrum” (43, 44) autistic development and a similar range across DQ.

A possible explanation for these results is that the intervention simply reduces arousal or anxiety and that this might affect ADOS scores. This may well partly be the case (for instance with the level of sensory and repetitive behaviours, that are sensitive to arousal states); but this is insufficient to explain the long-term sustained effects, and anyway begs the question of *why* the arousal reduces. More profoundly I would argue that if we change the fundamental and early sense of the child’s connection, acceptance, belonging, and being, within and accepting neurodiversity, then outcomes related to social motivation, engagement, and communication will predictably be altered. And since these latter aspects constitute core aspects of the phenotype as we currently understand and define it, then, to that extent, the phenotype is changed. This malleability then suggests something about the autistic phenotype as a “*state*” phenomenon—a state that is emergent under certain conditions and that can subside under others. This is not to imply an absence of other difficulties (47) but it points to something in the epistemology of the condition. Another inference could be that the autistic phenotype as measured like this is not actually the irreducible core “difference” experienced within neurodiversity—that the core phenotype lies somewhere else behind. I would be very open to this account, which I explore further below. But the relative malleability of part of the phenotype as measured here (and as encoded in the current nosology) opens up to another profound re-framing, that of autistic states within a transactional context—to which I now turn.

## Autism as transactional

With this evidence, we can now do something new to enlarge the nature of the complex system that we are describing within autism. Whereas traditionally within developmental science, the object of description has been the individual developmental trajectory (autism as an individual condition), and these dyadic intervention studies, along with the developmental theory that underpins them, enlarge this to include *both the individual and the immediate social environment in transactional relation*. This is not a new idea in developmental science generally; in both Winnicott’s famous formulation that “there is no such thing as a baby” (48), and Bowlby’s theory of the “goal-directed partnership” within early relatedness (49), there is an implicit recognition of the interpersonal context within which any individual development operates—an idea formalised in Sameroff’s transactional theory (50). However, traditionally autism, partly because of its high heritability, has been considered more from an essentialist rather than a transactional position. My aim is here to bring autism/neurodiversity into this transactional/developmental domain; a paradigm shift in the context of much previous theory and research.

In our intervention model above, we are essentially perturbing this *interpersonal dyadic early relational system around the child*; we make the perturbation by initiating a change in adult responsiveness to child communications, finding that perturbing the interpersonal system in this way has predictable effects on the child dyadic response according to well-described transactional dynamics within developmental science in neurotypical development. We show that such transactional dynamics are as applicable to neurodivergence and neurodifference as to neurotypicality. Further, in a way that seems consonant to that described in normative developmental theory, the child appears to internalise that dyadic experience into an acquired ability and *intra*personal dynamic that allows generalisation out into other interpersonal contexts. This is the dynamic underpinning social development in neurotypical children and the generalisation of acquired social skills into social abilities across contexts independent of immediate contingencies. In our work, we show that *intra*personal dynamic is also seen within autistic development (in contra-distinction to frequent assumptions that autistic children find it hard to generalise acquired skills across context).

Such a transactional framing of autism is consistent with normative social development and social development theory. The prototypical autism phenotype is in this sense not solely within-child phenomena, it is also partly a transactional phenomenon, which is in itself emergent in development depending on the characteristics of the child’s neurodivergence and immediate relational and physical environment. Not only is the course of brain maturation itself likely influenced by such an experienced environment (51); but development and identity are co-constructed, not only interpersonally but also socially. Here this developmental account converges with the autistic community’s advocacy around social adaptation and the social model of autism (52); although for a complete transactional model one needs to articulate both poles—the particular quality and characteristics of the neurodivergence as well as the characteristics of the environment, whether interpersonal or social.

This element of malleability in the outcome of autism phenotype is sufficient to show that it is not simply pre-formed or a mechanical translation of heritable probability or brain development; to an important extent, it is the developmental outcome of the neurodivergent brain, mind, and body as it grows in the transaction within its inter-personal and material environment. (An extreme case for the effects of environmental perturbation is also well-made from the results of investigations into the “natural experiment” of environmental perturbation consequent on extreme social deprivation within institutions. As it is now well-attested, such environmental conditions, if prolonged beyond 6 months in early infancy/childhood, can result in a social development homologous with autism (53), and indeed meet current criteria for the phenotype) (54).

### Steps towards a transactional model of autism and its development

For a developed transactional model of autistic development, therefore, I see no reason to discard the value and utility of a categorical behavioural phenotype. However, we can reframe the dimensional processes that go into its formation by adding the *inter*personal processes of the environmental transaction to the *intra*personal processes around heritable brain development, interactive specialisation, and neurodivergence. Two decades of “babysibs” longitudinal neuroscience work have not identified specific discrete markers of autism emergence, but rather more general perturbations in many aspects of the developing brain system that are linked to later autism (7, 8). In such a complex system, experimental perturbation of this kind through intervention can be a royal road to understanding specificity and causation in the developmental process.

The tradition of individual difference psychology (55) investigated the nature of variation in distributed biological (including neural), physical, and psychological traits, and the interplay between such trait variation and consequent environmental transactions and adjustments in producing developmental outcomes. From early temperament theory and research (56) came the related notion of “goodness of fit”; developmental outcomes were found to be crucially impacted by the quality of these observed and experienced transactions rather than simply the intrinsic properties of trait or biological variation—a theory elaborated in the transactional model (50). The developmental model proposed here applies these transactional theory ideas to neurodiversity—making it an extension, or special case, of individual difference theory, but stretching its envelope and explanatory power.

Logically from this, the constituent elements of such a transactional developmental model would need to include the identification of: (i) the specific characteristics of neurodiversity/neurodifference in the first few years of life; (ii) the interactional consequences and experiential responses of such specific neurodivergence; (iii) the consequent evolution of an outcome behavioural phenotype through early development; (iv) establishing causal influence between these reciprocal elements through experimental perturbation studies; (v) building and testing a developmental model by integrating (i)–(iv). Table 2 outlines some theory and current evidence for each of these steps.

**TABLE 2:** Elements of a proposed transactional theory transactional model of autism.

Model element	Current theory and evidence	Comments
***1.*** Early neurodivergence	Difference in visual response to eye gaze from 2 months (57), to speech sounds from 4 months (58), altered trajectories of social engagement with preserved attention to faces but reduced triadic attention to objects from 2 to 3 months, predicting social−communication skills at 2 years (59). These differences in perceptual organisation noted in different modalities later in the first year (60).	Preliminary evidence and these changes are subtle. No intervention experiments yet accomplished at this age to identify causal influence or potential for change.
***2.*** Interaction consequences of early neurodivergence	High autism likelihood (HL; siblings of autism probands) show relative increase in parent directiveness from 7 months compared to low likelihood (LL) infant-parent dyads, associated with altered visually evoked potentials in the infant (61). By 14 months the parent differences joined by observed infants alterations in social engagement and affect sharing with the adult; these infant observations predict autism emergence at 3 years in the cohort (39).	Since this dyadic interaction is itself a dynamic system, it can in some dyads re-organise into something itself less mutual and self-correcting, experienced often by parents’ account as despair or disengagement on the one hand or anxiously directive on the other. This interaction dynamic is the proximal target of early dyadic intervention (28, 40, 43).
3. Phenotypic outcome trajectories	Emerging group differences in behaviour between HL and LL infants in the latter part of the first year, involving dyadic social engagement, flexibility of attention, and integration between verbal and non-verbal communication, vocalisation and gesture, predict later autism development and suggest gradually emergent autistic trajectories (7, 8).	In the second year, Mottron et al. (12) postulate a more definitive threshold (bifurcation) event often signalled by abrupt skills regression, which they link to a decisive shift in the infant from a social to a non-social perceptual bias and resulting developmental disruption. This is postulated to further re-organise over into the (prototypical) pattern of phenotypic autism seen by the later 2nd and 3rd years.
4. Evidence from perturbation (intervention) experiments about causal influence	Re-orientation of parental focus and response to the child in therapy re-establishes reciprocal social interchange, with increased child social initiations and engagement [evidenced by video coding (27, 28, 43, 44)]. This mitigates the interaction consequences in section 2 above and is causally associated with improved social functioning beyond the dyad (30, 31) as well as increased reported connectedness and relatedness in parent’s experience of the child (33). This increased social orientation and engagement is sustained after the intervention period in subsequent development (43, 44)—and sufficiently in Whitehouse et al (44) to reduce outcome developmental characteristics below a prototypical autism threshold. Increased child social engagement and communication initiation is also the origin of the sustained intervention effect on outcome symptoms from pre-school to mid-childhood (29, 32).	Neurodivergent infant and child social engagement is a function of transactional experience, rather than “social avoidance” being an intrinsic part of the phenotype. The therapy arguably results in an internal rebalancing of social and object focus with increased social behaviours and decreased object focused routines. The child retains neurodifference but arguably to a less distressing and impairing degree and better able to take advantage of social life. This evidence thus supports a *relative* malleability of processes within autistic development—and the idea that the prototypic phenotype can both emerge and relatively “sub-merge.” While “bifurcation” suggests a linear and irreversible process, emergence has something more of a state quality: a combination of distributed dimensional dynamics induces a state change with emergent characteristics, which can reverse.

## Autistic states and irreducible difference—The role of phenomenology

I hope the evidence will be clear from the above account that there is an element of malleability in the early emergent phenotype of autism. This is important at the practical level of providing early evidenced support for diagnosed autistic children and those infants who are at increased likelihood of an autistic trajectory. The intervention science suggests that this support will benefit these children and their families, not just at a phenotype level but in terms of wellbeing and confidence, and we are now in a position as this intervention science has proceeded, to advocate now for a practical and evidenced integrated early care pathway within health systems (62).

At a more epistemological level, a transactional approach identifies some phenotypic malleability, but there remains behind these undoubtedly irreducible aspects of neurodiversity and experience; about which there is no evidence for or intention here to “remove” or “eradicate” through intervention or support. Further progress towards refining understanding of this more irreducible part of the phenotype will require new strategies and complimentary measurement. This will entail centrally at this point in my view an approach to the phenotype from the “inside-out” through phenomenology. It is an extraordinary lacuna to date in mainstream autism science that there is no *systematic autistic phenomenology*. The prototypical autism phenotype measured and analysed above has always been characterised externally from observed behaviour (in common with many developmental conditions from early childhood)—a fact that has certainly limited theorising and has naturally led to criticism from those with lived experience and others that much about the current phenotypic description objectifies them. While sympathetic observation and careful neuroscience can still increase empathic understanding, the time has come for this to be complimented by phenomenology and systematic data from lived experience. This is partly an ethical imperative, but it will also fill in a key gap scientifically in understanding autism and a dynamic systems perspective on its emergence. We have much narrative information already from the often extraordinary and rich accounts that have been written by autistic people and parents of autistic children. But a systematic exploration of such phenomenology using shared qualitative and quantitative methods will add much more generalisability to this existing information and allow comparison with other areas, for instance, of neurotypical experience. There have been increasing calls for more systematic attention to the lived phenomenology of autism (63, 64), and some early work has begun (65). The perspective of the development of the experiencing mind if systematically done will clarify much in developmental science and direct the focus of research going forward. For instance, gaining a richer and more general understanding of the experienced autistic sensorium within different environments, the experience of space and time and attentional focus, will all be central to a fuller phenotype description and potentially valuable for the direction of approach in autism neuroscience. Systematic work by and with autistic people will be core to this approach; they would be at the centre of a phenomenologically informed phenotype—and the new measures needed to describe it. Such joint work joint work may act as a further practical bridge through action between neurodiversity, clinical and research perspectives on “what is autism.”

I have argued that, for further evolution of the developmental science of autism at this point, we will need to elaborate a more nuanced and transactional account of what it means to be autistic and how autism becomes itself; and I have evidenced how information from clinical trials can contribute to that. Moreover, in doing so, we may not wish to dispense with autism as an entity, despite its paradoxes; and may want to consider the prototypical version that Mottron advocated as one pole of thought and action. I intend this article to outline one way to do this.

## Ethics statement

The studies involving human participants were reviewed and approved by Greater Manchester Research Ethics Committee. Written informed consent to participate in this study was provided by the participants’ legal guardian/next of kin.

## Author contributions

The author confirms being the sole contributor of this work and has approved it for publication.

## Footnotes

1
www.nimh.nih.gov/research-priorities/rdoc/index.shtml
